# The forgotten middle: How moderate self-efficacy amplifies the threat of AI through job insecurity

**DOI:** 10.3389/fpsyg.2025.1734254

**Published:** 2026-01-12

**Authors:** Xinrui Liu, Zijian Ye

**Affiliations:** 1 School of Business and Trade, Anhui Wenda University of Information Engineering, Hefei, China; 2 School of Business, Sunway University, Kuala Lumpur, Malaysia; 3 Faculty of Business and Economics, Universiti Malaya, Kuala Lumpur, Malaysia

**Keywords:** artificial intelligence, job insecurity, self-efficacy, suppression-based mediation, curvilinear moderation, employee performance

## Abstract

**Introduction:**

Artificial intelligence (AI) has sparked a paradox in organizational behavior research: while it promises productivity gains, it simultaneously generates psychological strain and inconsistent performance outcomes.

**Methods:**

Drawing on Conservation of Resources (COR) theory and technology empowerment theory, this study investigates how AI adoption affects employee job performance through job insecurity and how self-efficacy shapes this relationship in a nonlinear way. Using multi-source paired data from 392 employees and their supervisors in China’s cross-border e-commerce sector, the study tests a suppression-based mediation model.

**Results:**

The results reveal that AI’s positive technological empowerment is fully offset by its negative psychological threat, forming a suppression structure. Job insecurity mediates the relationship between AI application and performance, while self-efficacy moderates this effect in an inverted U-shaped manner—employees with moderate self-efficacy experience the highest insecurity and the strongest indirect negative effect.

**Discussion:**

These findings advance COR theory by conceptualizing self-efficacy as a finite resource and highlight how psychological mechanisms determine whether AI empowers of undermines employees.

## 1 Introduction

Artificial intelligence (AI) has ignited a striking paradox in contemporary organizations—while it promises unprecedented productivity gains, it simultaneously triggers psychological strain and performance declines. Despite record-breaking investments and widespread adoption, empirical findings remain inconsistent: some studies show AI boosts output and decision quality (Brynjolfsson and McAfee, 2017; Noy and Zhang, 2023), whereas others reveal declines in motivation, engagement, and wellbeing (Grewal et al., 2024). This inconsistency forms what we term the AI–performance paradox—a tension between technological empowerment and psychological threat that challenges both scholars and practitioners. Understanding why and when AI improves or impairs performance is no longer an incremental question but a theoretical imperative.

Among the various psychosocial responses to AI, *AI-induced job insecurity* has emerged as one of the most salient (Lee et al., 2018; Shoss, 2017). Job insecurity refers to an individual’s perceived threat to the stability of their employment (De Witte, 2005), but in the age of AI, this concern extends to an even deeper form—*occupational insecurity*—where employees fear that entire professions could become obsolete (Autor, 2015). Empirical studies confirm that such fears are not unfounded. Acemoglu and Restrepo (2020) demonstrated that each additional industrial robot per thousand workers reduces employment by 0.2 percentage points and depresses wages. In this context, fear of technological replacement has become a critical psychosocial cost of digital transformation.

These anxieties have meaningful organizational consequences. High levels of job insecurity are consistently linked with lower job satisfaction, reduced engagement, burnout, and higher turnover intentions (Sverke et al., 2002; Cheng and Chan, 2008; Sverke et al., 2019). Moreover, organizations that fail to manage employees’ psychological adaptation to AI may erode valuable human capital and undermine the very productivity gains they seek to achieve (Hobfoll, 1989, 2001). In other words, neglecting the *human side of AI transformation* risks converting technological advantage into human vulnerability.

This paradox gives rise to a pressing theoretical question: under what conditions does AI adoption enhance or hinder employee performance? While previous studies have documented AI’s direct benefits for performance (Rodriguez et al., 2025) and its negative psychological outcomes (Kellogg et al., 2020; Wood et al., 2019), these streams have evolved largely in isolation. The literature remains fragmented, with limited integration between technological and psychosocial perspectives (Makarius et al., 2020; Jöhnk et al., 2021). A more holistic framework is needed to reconcile these opposing effects and explain individual differences in employees’ reactions to AI.

To address this gap, the present study introduces *self-efficacy* (Bandura, 1997) as a critical boundary condition shaping employees’ cognitive and emotional responses to AI. Self-efficacy—the belief in one’s ability to organize and execute actions to achieve desired goals—plays a central role in coping with technological change (Compeau and Higgins, 1995; Judge and Bono, 2001). While higher self-efficacy typically promotes adaptive behavior, evidence suggests that its effect may not be linear. Excessive confidence can lead to complacency and reduced performance (Vancouver et al., 2001), whereas moderate levels of self-efficacy may heighten vigilance and stress. This study thus posits that self-efficacy exerts a curvilinear (inverted U-shaped) moderating effect on the relationship between AI adoption and job insecurity.

This study contributes to theory by revealing that self-efficacy—a central construct in social cognitive theory—is not an endlessly protective resource under technological threat. Consistent with Conservation of Resources (COR) theory (Hobfoll, 1989, 2001), psychological resources are finite; when facing AI-driven uncertainty, excessive confidence may backfire. Although alternative frameworks such as the Job Demands–Resources model (Bakker and Demerouti, 2007) or the Transactional Theory of Stress (Lazarus and Folkman, 1984) could also explain employees’ responses to AI, COR theory provides a more comprehensive explanation of resource gains and losses triggered by technological disruption. Unlike JD–R, which primarily focuses on job demands and resources, COR captures the existential loss of valued objects (e.g., employment stability and career continuity). This makes COR particularly suitable for theorizing AI-induced job insecurity as a deep psychological threat rather than merely an increased job demand. We advance COR theory by demonstrating that self-efficacy exhibits a curvilinear (inverted U-shaped) moderation in the AI context, redefining it from a linear buffer to a finite and sometimes self-defeating resource.

The next section presents the theoretical framework and hypothesis development.

## 2 Theoretical background and conceptual framework

### 2.1 Literature review

Artificial intelligence (AI) has fundamentally transformed how organizations function, influencing both efficiency and employee experience. Over the past decade, scholars have increasingly recognized AI not merely as a technological tool but as an institutional force reshaping organizational structure, work roles, and performance logic (Brynjolfsson and McAfee, 2017). AI’s impact unfolds along two principal pathways—automation and augmentation (Autor, 2015; Bessen, 2019). The automation path allows machines to replace repetitive and rule-based tasks, thereby reducing human labor costs, while augmentation enables employees to extend cognitive capabilities through AI-based decision support systems.

A substantial body of empirical research confirms that, particularly in knowledge-intensive industries, AI significantly enhances individual and organizational productivity (Brynjolfsson et al., 2023; Noy and Zhang, 2023). The task–technology fit (TTF) theory posits that technology improves performance when its functionality aligns with task requirements (Goodhue and Thompson, 1995). Studies in sales and service contexts support this notion, showing that technology use enhances information integration and task reconfiguration, which in turn improve performance outcomes (Ahearne et al., 2008; Hunter and Perreault, 2007).

Recent advances in generative AI (GenAI) have expanded this research frontier. Unlike predictive AI, GenAI systems can create new content and support complex communication, generating measurable performance gains in domains such as sales, customer service, and content creation (Paschen et al., 2020; Mikalef et al., 2023). For instance, Rodriguez et al. (2025) found that GenAI significantly improves sales effectiveness and administrative efficiency, with managerial support amplifying these effects. Similarly, experimental evidence from Brynjolfsson et al. (2023) revealed that ChatGPT use improved textual quality by 37% and work speed by 56%, suggesting that AI’s empowering effects stem from automation, cognitive augmentation, and informational transparency (Singh et al., 2019).

However, the rapid diffusion of AI has also generated significant psychological stressors, especially job insecurity. Drawing on Conservation of Resources (COR) theory (Hobfoll, 1989, 2001), scholars argue that individuals experience stress when they perceive potential or actual loss of valued resources such as employment, skills, or status. As AI permeates organizational processes, employees’ concerns about technological substitution and skill obsolescence intensify (Shoss, 2017; Lee et al., 2018). Numerous studies have established that job insecurity undermines job satisfaction, organizational commitment, and task performance (De Witte, 2005; Cheng and Chan, 2008; Sverke et al., 2019). Labor economics evidence further confirms these concerns: each additional robot per thousand workers reduces local employment by 0.2 percentage points (Acemoglu and Restrepo, 2020), and wage growth slows significantly in highly automated sectors (Frey and Osborne, 2017; Autor and Dorn, 2013).

These findings underscore AI’s dual-edged nature—its capacity to empower and threaten simultaneously. While AI enhances efficiency, it also triggers cognitive overload and perceived control loss, leading to technostress (Tarafdar et al., 2007; Ayyagari et al., 2011). Later studies found that technological complexity and excessive demands can reduce job engagement and performance (Tarafdar et al., 2015). Algorithmic management further amplifies these psychological pressures through performance monitoring and quantification, provoking defensive reactions and emotional exhaustion (Kellogg et al., 2020; Wood et al., 2019). Consequently, AI adoption can yield a paradoxical outcome—enhancing productivity while simultaneously undermining psychological wellbeing (Grewal et al., 2024).

The magnitude of AI’s effects depends strongly on individual and contextual factors. Among these, self-efficacy has been identified as a key psychological resource influencing adaptation to technology (Bandura, 1997; Compeau and Higgins, 1995; Judge and Bono, 2001). High self-efficacy employees tend to interpret AI as a support tool rather than a threat and thus engage in proactive learning and integration (Makarius et al., 2020). Meta-analytic findings confirm a robust positive relationship between self-efficacy and performance (Judge et al., 2005). Yet, this relationship may not always be linear. Excessively high self-efficacy can lead to overconfidence bias and complacency, ultimately reducing performance (Vancouver et al., 2001). This curvilinear pattern aligns with the classic Yerkes–Dodson law Yerkes and Dodson (1908), which suggests that moderate levels of arousal or challenge lead to optimal performance.

Beyond individual traits, organizational context also shapes AI outcomes. The Technology Acceptance Model (TAM) emphasizes perceived usefulness and ease of use as central determinants of technology adoption (Davis, 1989; Venkatesh and Davis, 2000; Venkatesh and Bala, 2008). Organizational support and managerial attitude can moderate these perceptions by enhancing psychological safety and training opportunities (Eisenberger et al., 1986; Rodriguez et al., 2025). Research on AI readiness further highlights leadership and organizational culture as critical enablers of successful AI implementation (Jöhnk et al., 2021).

Despite the growing research on AI empowerment and technostress, prior studies tend to examine these perspectives separately, resulting in fragmented insights. Little is known about how empowerment and threat may coexist or interact, leaving a theoretical gap that the present study aims to address. Integrating these perspectives, AI’s impact on performance involves two opposing pathways: (1) a technological empowerment effect, where AI enhances performance by increasing efficiency and decision quality, and (2) a psychological threat effect, where AI-induced insecurity undermines motivation and engagement. The coexistence of these mechanisms constitutes a suppression-based mediation structure (MacKinnon et al., 2000; Zhao et al., 2010). The net effect of AI thus depends on the balance between empowerment and threat, influenced by employees’ self-efficacy and organizational context.

### 2.2 Conceptual model and hypotheses development

Building on the preceding review, this study develops and empirically tests a moderated suppression-based mediation model linking AI application, job insecurity, self-efficacy, and job performance. This framework integrates technology empowerment theory and COR theory to explain both the positive and negative pathways through which AI affects employees’ performance outcomes.

H1: AI application has a positive direct effect on employee job performance (*technological empowerment effect*).

H2: AI application positively predicts employees’ job insecurity.

H3: Job insecurity negatively predicts employees’ job performance.

H4: Job insecurity mediates the relationship between AI application and job performance, forming a *suppression-based mediation* structure where the indirect effect is negative.

H5: Self-efficacy exerts an inverted U-shaped moderating effect on the relationship between AI application and job insecurity, such that the positive impact of AI on job insecurity is strongest at moderate levels of self-efficacy.

H6: The indirect effect of AI application on job performance via job insecurity varies with employees’ self-efficacy, forming a curvilinear *conditional indirect effect* that is strongest at moderate self-efficacy levels.

**FIGURE 1:**
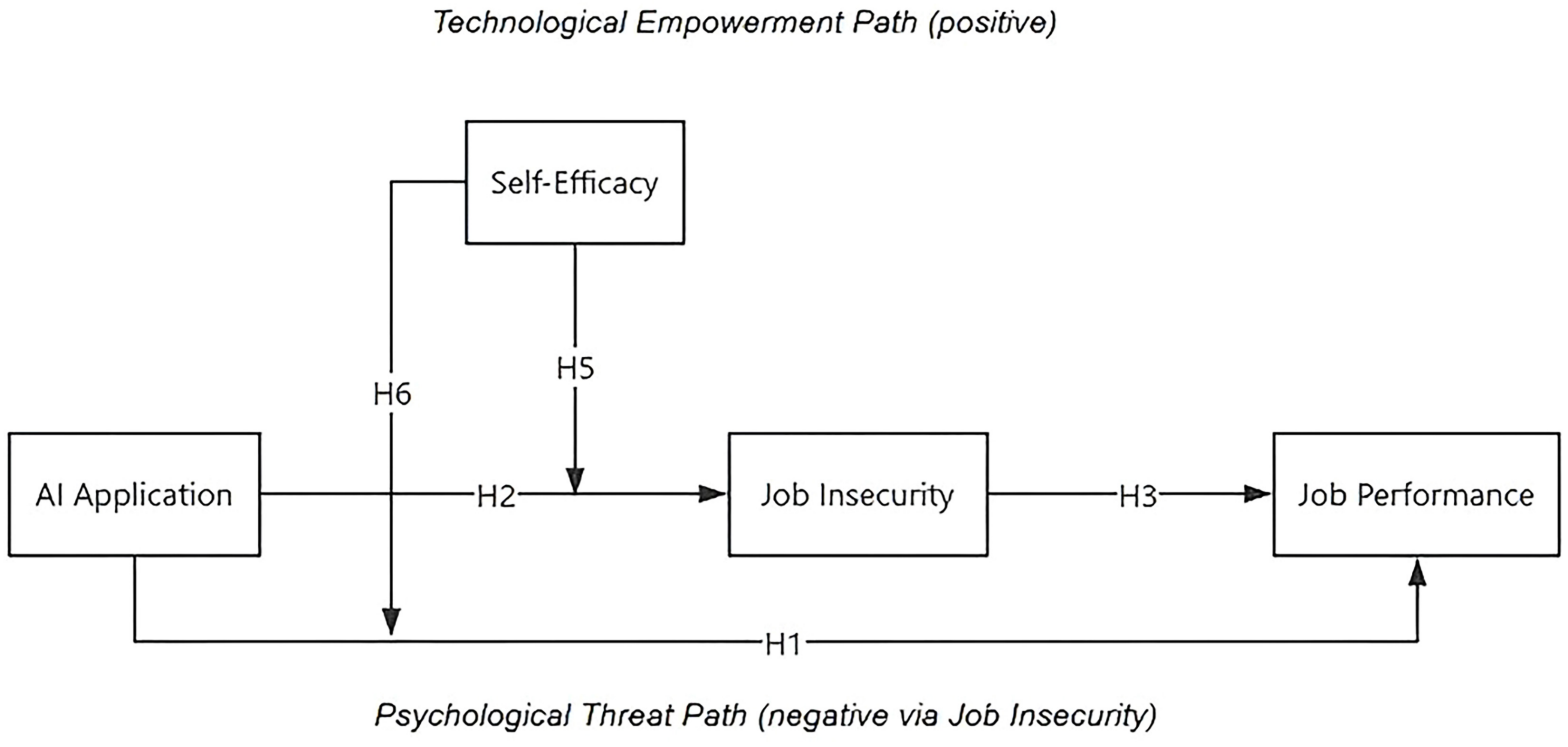
Conceptual model. The model depicts two opposing mechanisms: the Technological Empowerment Path (positive) and the Psychological Threat Path (negative via Job Insecurity). The coexistence of these opposite pathways produces a suppression structure at the overall level.

Figure 1 illustrates the overall conceptual model integrating these hypotheses. Through this framework, the study seeks to move beyond the simplistic “AI is good or bad” dichotomy and instead explain when and why AI adoption produces divergent psychological and performance outcomes among employees.

## 3 Materials and methods

### 3.1 Sample and procedure

We employed a multi-source, cross-sectional survey in 45 Chinese cross-border e-commerce firms that routinely use AI tools in daily operations (e.g., automated product listing, algorithmic pricing, chatbot-based service). Employees completed measures of AI application, job insecurity, self-efficacy, and demographics; their direct supervisors subsequently rated employees’ job performance. Data were collected between July and August 2025 using both online and on-site distribution. In total, 550 employees were invited; 421 provided usable responses (76.5% response rate). Matched supervisor ratings were obtained for 392 employees, yielding the final analytic sample (71.3% matched retention). To protect confidentiality, employee and supervisor questionnaires were linked via anonymous codes. Each supervisor rated no more than five direct reports and was instructed to evaluate typical performance over the past six months. All participants gave informed consent.

### 3.2 Measures

Unless otherwise noted, all variables were assessed on seven-point Likert scales (1 = strongly disagree, 7 = strongly agree). English items followed published scales; Chinese items were adapted using a translation–back-translation procedure. All instruments are widely used research measures in the public domain or used with permission from the original authors.

#### 3.2.1 AI application

Artificialintelligence use was measured with the Information Systems Use scale (Sun and Teng, 2012). The ISU scale was chosen because it captures the frequency, depth, and scope of technology-enabled work activities, dimensions that align with how AI tools are operationalized in cross-border e-commerce contexts. Although originally developed for general information systems, its behavioral usage focus makes it suitable for assessing AI-supported work routines. ISU adapted to reflect AI-enabled functions in e-commerce (12 items across daily operations, decision support, and team collaboration). Sample items include: “I use AI-powered tools to support my daily work tasks,” “AI systems assist me in making better decisions,” and “Our team uses AI to enhance collaboration and coordination.”

#### 3.2.2 Job insecurity

Job insecurity was assessed with the seven-item scale by Hellgren et al. (1999), capturing quantitative and qualitative dimensions. Items tapping qualitative insecurity were reverse-coded. Sample items: “I feel uncertain about the continuity of my job” and “Important aspects of my job may change for the worse.”

#### 3.2.3 Self-efficacy

General self-efficacy was measured with the 10-item General Self-Efficacy Scale (Schwarzer and Jerusalem, 1995). A sample item is: “I am confident that I can deal efficiently with unexpected events.”

#### 3.2.4 Job performance

Supervisors rated employees’ performance using a 10-item scale based on the task–contextual performance framework (Borman and Motowidlo, 1993), with five task-performance items (e.g., “This employee meets performance standards and productivity goals”) and five contextual-performance items (e.g., “This employee willingly helps colleagues with heavy workloads”).

We controlled for gender, age, education, and organizational tenure, which are commonly associated with performance outcomes.

### 3.3 Empirical analysis

#### 3.3.1 Pilot study and scale validation

A pilot survey yielded 80 valid responses (response rate = 89.9%). All scales demonstrated satisfactory internal consistency (Cronbach’s α > 0.70; Nunnally, 1978). Convergent validity was supported by average variance extracted (AVE > 0.50; Fornell and Larcker, 1981) and factor loadings above.70 (Gefen and Straub, 2005). Preliminary PLS analyses and correlations conformed to expectations. Based on supervisor feedback, we refined instructions, enforced anonymous code-matching, and limited each supervisor to rating at most five subordinates.

#### 3.3.2 Main study: reliability, validity, and descriptive statistics

Table 1 reports construct reliability and validity. Cronbach’s α exceeded.70, composite reliability (CR) exceeded.80, and AVE exceeded.50 for all constructs. The square root of AVE for each construct was larger than its inter-construct correlations, supporting discriminant validity. Bivariate correlations showed that AI Application correlated positively with Job Insecurity (*r* = 0.509, *p* < 0.01), Job Insecurity correlated negatively with Job Performance (*r* = −0.650, *p* < 0.01), and Self-Efficacy correlated positively with Job Performance (*r* = 0.660, *p* < 0.01). Table 2 summarizes sample characteristics (*N* = 392): 59.2% female; 85.8% held a bachelor’s degree or higher; 91.8% were aged 40 or younger; tenure and firm size distributions are reported.

**TABLE 1:** Results of reliability, convergent, and discriminant validity analyses.

Construct	α	CR	AVE	√AVE	1	2	3	4
1. AI application	0.891	0.874	0.62	**0.79**	1	–	–	
2. Job insecurity	0.878	0.865	0.59	**0.77**	0.509**	1	–	–
3. Self-efficacy	0.839	0.851	0.57	**0.84**	0.596**	−0.713**	1	–
4. Job performance	0.722	0.781	0.55	**0.81**	0.493**	−0.650**	0.660**	1

Diagonal values (in bold) represent the square roots of AVE.

*p < 0.05,

**p < 0.01.

**TABLE 2:** Sample characteristics (*N* = 392).

Factors	Category	Frequency (*N* = 392)	Percentage (%)
Gender	Male	160	40.8
	Female	232	59.2
Age	≤20 years	2	0.5
	21–30 years	164	41.8
	31–40 years	194	49.5
	41–50 years	22	5.6
	51–60 years	10	2.6
Education	High school or below	16	4.1
	Associate degree	40	10.2
	Bachelor’s degree	246	62.8
	Master’s degree or above	90	23
Years of work experience	≤1 year	18	4.6
	1–3 years	50	12.8
	3–5 years	96	24.5
	5–10 years	136	34.7
	≥10 years	92	23.5
Firm size	<50 employees	18	4.6
	50–200 employees	100	25.5
	200–500 employees	150	38.3
	500–1,000 employees	64	16.3
	>1,000 employees	60	15.3
Total		392	100

Percentages may not total 100% due to rounding.

#### 3.3.3 Analytic strategy

We tested mediation and moderated-mediation hypotheses using the PROCESS macro (Hayes) in SPSS with 5,000 bootstrap resamples. Continuous predictors were mean-centered. For suppression-based mediation (H1–H4), we estimated the direct path from AI Application to Job Performance (H1), the path from AI Application to Job Insecurity (H2), and from Job Insecurity to Job Performance (H3). Indirect effects were evaluated via bias-corrected bootstrap confidence intervals (H4).

To assess the curvilinear moderation of Self-Efficacy (H5), we included both the linear and squared terms of Self-Efficacy and their interactions with AI Application in predicting Job Insecurity. Conditional indirect effects across Self-Efficacy levels (−1 SD, mean, +1 SD) were then computed to test H6.

### 3.4 Results overview for hypothesis tests

Table 3 summarizes the mediation path analysis. Results indicate that AI Application positively predicted Job Insecurity (β = 0.416, *p* < 0.001; supporting H2), which in turn negatively predicted Job Performance (β = −0.528, *p* < 0.001; supporting H3). Regarding the direct pathway, the effect of AI Application on Job Performance (H1) was positive but non-significant (β = 0.072, *p* = 0.241). In contrast, the indirect effect via Job Insecurity was significantly negative (Indirect Effect = −0.220, 95% CI [−0.347, −0.114]), supporting H4. This pattern—where a significant negative indirect effect coexists with a non-significant direct effect—is consistent with a suppression structure. Notably, the reported indirect effect (−0.220) represents the completely standardized effect size, which qualifies as a medium effect according to Cohen’s conventions. Furthermore, the full model including both direct and indirect paths explains approximately 31% of the variance in job performance (R^2^ = 0.31).

**TABLE 3:** Mediation path analysis (H1–H4).

Outcome/predictor	β	SE	t	*P*	R^2^	Support
Model 1: job insecurity (M)	–	–	–	–	0.17	–
AI application (H2)	0.416	0.081	5.13	<0.001	–	√
Model 2: job performance (Y)	–	–	–	–	0.31	–
Job insecurity (H3)	−0.528	0.074	−7.14	<0.001	–	√
AI application (H1)	0.072	0.061	1.18	0.241	–	×
Indirect effect (H4)	Effect	BootSE	LLCI	ULCI	Type	
AI→ insecurity → performance	−0.22	0.059	−0.347	−0.114	Medium (0.22)	√

Indirect effects tested with 5,000 bias-corrected bootstrap samples. Significance determined when 95% confidence interval does not include zero.

**FIGURE 2:**
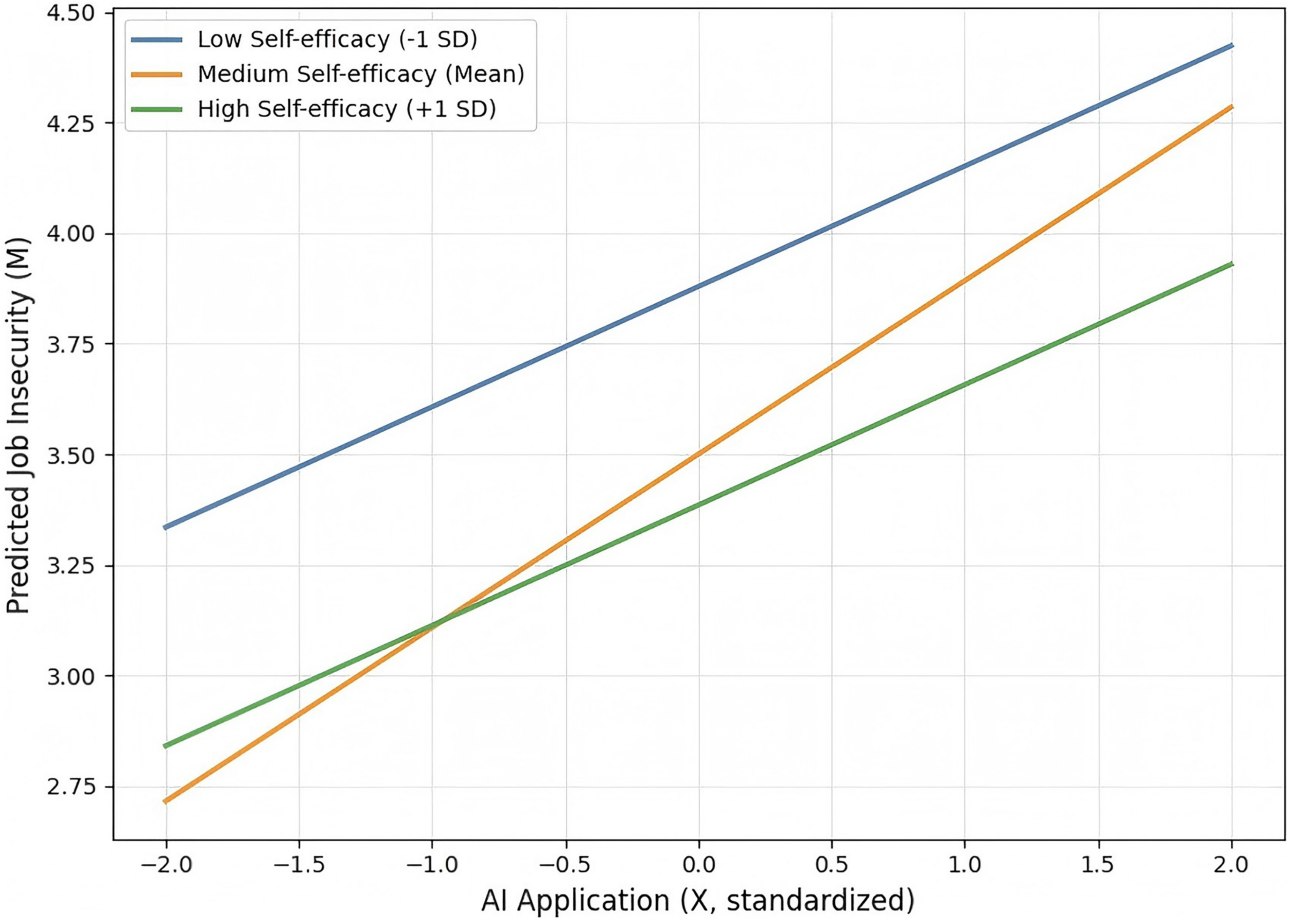
Interaction between artificial intelligence (AI) application and self-efficacy on job insecurity. The plot displays simple slopes. The steepest slope at the mean level of self-efficacy, compared to the flatter slopes at low and high levels, visualizes the inverted U-shaped moderating effect.

For the curvilinear moderation (Table 4), the interaction between AI Application and the squared term of Self-Efficacy was significant (β = −0.184, *p* = 0.032), indicating an inverted U-shaped moderation: the positive effect of AI on Job Insecurity peaked at moderate levels of Self-Efficacy (H5 supported). Figure 2 illustrates how the magnitude of AI’s impact on job insecurity varies as a function of self-efficacy. Specifically, the positive effect intensifies from 0.295 at low self-efficacy to a peak of 0.439 at moderate self-efficacy, before diminishing to 0.280 at high self-efficacy. This distinct “low–high–low” pattern in slope strength confirms the hypothesized inverted U-shaped moderating effect.

**TABLE 4:** Curvilinear moderation of self-efficacy on the relationship between artificial intelligence (AI) application and job insecurity (H5).

Predictor	β	SE	t	*P*
AI application	0.392	0.078	5.03	<0.001
Self-efficacy	−0.247	0.086	−2.87	0.004
Self-efficacy^2^	0.132	0.056	2.36	0.019
AI application × self-efficacy	−0.094	0.061	−1.54	0.124

Dependent variable = Job Insecurity. Predictors mean-centered; 5,000 bootstrap samples. The significant interaction term for the squared moderator confirms an inverted U-shaped moderation.

**FIGURE 3:**
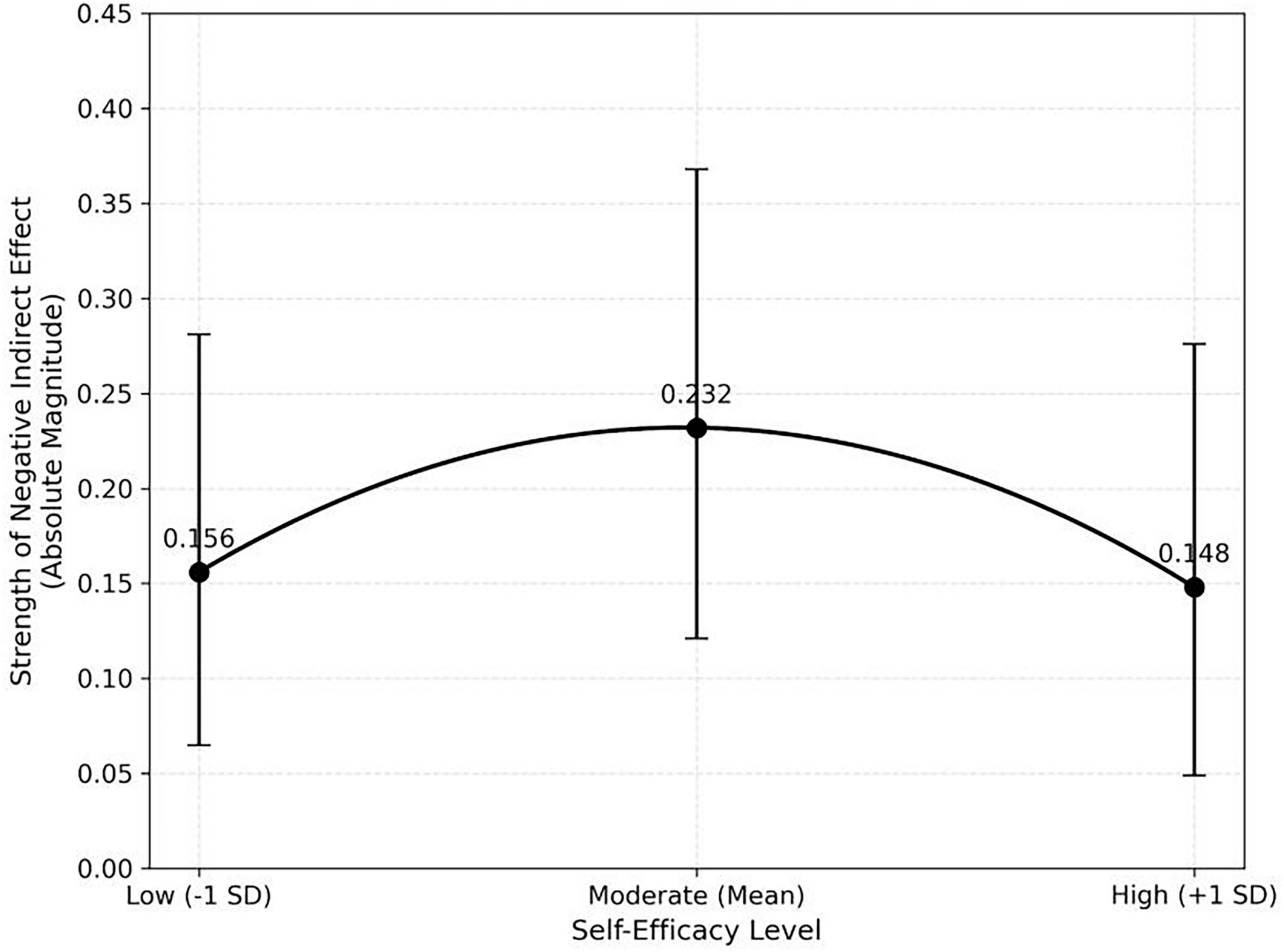
Strength of conditional indirect effects across levels of self-efficacy.

Conditional indirect effects (Table 5) were significant at low (Effect = −0.156, 95% CI [−0.281, −0.065]), mean (Effect = −0.232, 95% CI [−0.368, −0.121]), and high levels (Effect = −0.148, 95% CI [−0.276, −0.049]) of Self-Efficacy, with the strongest negative indirect effect at the mean level, supporting H6.It is important to note that while the indirect effects are statistically negative (indicating harm to performance), Figure 3 plots their absolute magnitudes to visualize the strength of this impact. By focusing on the magnitude (i.e., converting −0.232 to 0.232), the graph clearly reveals an inverted U-shaped trajectory. This visual transformation highlights that although the detrimental effect is universal, its intensity peaks at the moderate level of self-efficacy.

**TABLE 5:** Conditional indirect effects of artificial intelligence (AI) application on job performance via job insecurity at different levels of self-efficacy (H6).

Level of self-efficacy	Indirect effect	95% CI lower	95% CI upper	Supported
Low (−1 SD)	−0.156	−0.281	−0.065	√
Mean	−0.232	−0.368	−0.121	√
High (+1 SD)	−0.148	−0.276	−0.049	√

Conditional indirect effects estimated with 5,000 bias-corrected bootstrap resamples. All confidence intervals exclude zero, supporting moderated mediation (H6).

### 3.5 Ethics statement

The study complied with institutional ethical guidelines. Participation was voluntary, and respondents were assured of confidentiality and the exclusive academic use of data. Written informed consent was obtained from all participants. No personally identifiable information was stored alongside survey responses.

## 4 Discussion

To explain this inverted U-shaped pattern, we identify a “peak sensitivity” mechanism. Employees with low self-efficacy may experience a form of “learned helplessness” (Seligman, 1972); believing they cannot adapt to AI regardless of effort, they may psychologically disengage, resulting in paradoxically lower reported insecurity. Conversely, those with high self-efficacy perceive AI as a manageable challenge rather than a threat.

However, employees with moderate self-efficacy occupy a precarious middle ground. They possess enough competence to recognize the magnitude of the AI threat but lack the sufficient confidence to feel assured of their survival. This state of “cognitive dissonance”—caring about the outcome but doubting one’s success—generates the highest level of vigilance and anxiety.

### 4.1 Key findings

The results reveal several multilayered insights grounded in the specific context of China’s cross-border e-commerce sector.

First, AI application exerts a significant suppression-based mediation effect on job performance. The direct effect of AI on job performance (H1) was positive but statistically non-significant. According to Zhao et al. (2010), when the direct effect and the indirect effect are in opposite directions and only the indirect effect is significant, this pattern constitutes a suppression effect rather than full competitive mediation. This means that AI’s positive technological empowerment is fully masked by its negative psychological pathway through job insecurity, yielding an apparently neutral total effect. In other words, the non-significant direct effect is not a null result but a strong confirmation that opposing mechanisms—empowerment and threat—operate simultaneously and cancel each other out.

Conceptually, this suppression aligns with COR theory: the empowerment path adds resources (efficiency, control), whereas the insecurity path depletes resources (stability, certainty). The net-zero direct effect thus reflects dynamic resource equilibrium rather than the absence of influence (Hobfoll, 2011).

Second, self-efficacy exhibits a non-linear (inverted U-shaped) moderating role. Supporting Hypothesis 5, the positive relationship between AI application and job insecurity peaked at moderate self-efficacy levels. This challenges the traditional assumption that “the higher the self-efficacy, the better.” Low self-efficacy employees tend to avoid threats due to lack of confidence, whereas high self-efficacy individuals confront AI confidently and treat it as a challenge rather than a threat. Employees with moderate self-efficacy, however, are most sensitive—they recognize AI’s substitution potential but doubt their own coping ability, experiencing the highest insecurity. To unpack this non-linear pattern, three psychological mechanisms can be identified. First, employees with low self-efficacy tend to experience learned helplessness (Seligman, 1972): lacking confidence in their ability to adapt, they disengage from challenges and underreact to potential threats, resulting in a muted insecurity response. Second, employees with moderate self-efficacy occupy a peak sensitivity zone. They are competent enough to recognize AI’s substitution potential but not confident enough to feel in control; this cognitive conflict produces heightened vigilance and the strongest insecurity. Third, employees with high self-efficacy display overconfidence bias (Vancouver et al., 2001): they overestimate their ability to cope and therefore appraise AI as more of a challenge than a threat. This curvilinear pattern is consistent with the Yerkes–Dodson law that moderate arousal optimizes adaptation, whereas too little or too much undermines coping. In AI contexts, self-efficacy acts as a regulatory lens that shapes whether technological change is appraised as a challenge or a hindrance/threat (Cavanaugh et al., 2000).

Finally, this inverted U-shaped moderation transmits across the mediation chain. Conditional indirect effect analyses show that the negative indirect effect of AI on performance via job insecurity was strongest at moderate self-efficacy, compared to low and high levels. Thus, employees with moderate self-efficacy are the most psychologically vulnerable group under AI adoption and serve as the critical transmitters of its negative performance consequences.

### 4.2 Theoretical implications

This research offers three main theoretical contributions.

First, by empirically validating a suppression-based mediation mechanism, it provides a psychological explanation for the long-standing AI productivity paradox. The paradox that technological investment does not always translate into proportional productivity gains may stem from psychological mechanisms that offset technological benefits. Our findings show that AI’s effect on performance comprises a positive efficiency pathway and a negative psychological cost pathway. Ignoring the latter risks overestimating AI’s net benefits. Future task–technology fit and technology acceptance research should therefore incorporate psychological cost factors to better capture the full consequences of digital transformation (Goodhue and Thompson, 1995; Venkatesh and Davis, 2000).

Second, this study extends technostress theory by conceptualizing AI-induced job insecurity as representing the existential dimension of technostress—a distinct psychological strain driven by the fear of substitution rather than mere usage difficulty. While traditional technostress research primarily focuses on work overload or complexity (Tarafdar et al., 2007; Ayyagari et al., 2011), our findings reveal that AI represents a fundamental threat to professional survival. The significant positive relationship between AI use and job insecurity (β = 0.416) empirically validates this shift, demonstrating that in high-pressure e-commerce settings, the primary stressor is not the inability to use technology, but the threat of being replaced by it. This offers new evidence on the existential nature of technology-induced strain (Shoss, 2017; Lee et al., 2018).

Third, the study introduces a novel non-linear perspective on self-efficacy in AI-related contexts. Self-efficacy does not uniformly buffer against technological threats but rather shapes the sensitivity threshold of individuals’ responses. When facing disruptive technologies that may reshape occupational structures, self-efficacy determines not only the magnitude but also the sensitivity of reactions. This finding calls for a shift from the traditional linear buffering view of individual differences toward a non-linear sensitivity perspective in human–AI interaction research. Future research may embed AI-induced job insecurity within broader organizational psychology perspectives, such as occupational identity threat and adaptation-to-change models, to further expand the theoretical scope.

### 4.3 Practical implications

The findings of this study offer critical guidance for managers navigating the human side of AI transformation.

First, organizations should move away from generic training approaches and instead adopt tailored interventions that address specific employee characteristics. Our results indicate that employees with moderate self-efficacy are particularly sensitive to the threats posed by AI, often recognizing the challenge but lacking the confidence to adapt. To support this group, managers can implement adaptive training programs that focus on achieving small, incremental wins. By breaking down complex AI applications into manageable tasks, organizations can help these employees gradually accumulate mastery experiences, thereby shifting their mindset from anxiety to confidence.

Second, establishing a climate of psychological safety is crucial to resolving the suppression effect caused by job insecurity. Since the fear of replacement can obscure the productivity benefits of AI, it is important to separate AI implementation from the prospect of workforce reduction. Organizations can establish “AI sandboxes” which serve as safe experimental zones where trial and error are encouraged. This approach reduces the anxiety associated with making mistakes and reinforces the perception of AI as a tool for augmentation rather than performance monitoring.

Third, AI literacy initiatives should be broadened to emphasize the logic of human-AI collaboration rather than focusing solely on technical operation. To counteract the fear of technological substitution, leaders need to articulate the unique value of human skills, such as complex judgment and empathy, which AI cannot replicate. By clarifying how human agency complements algorithmic power, managers can assist employees in redefining their professional identities, ultimately balancing technological empowerment with psychological security.

Organizations should provide adaptive training, psychological safety interventions, or AI-literacy programs specifically targeting employees with moderate self-efficacy, who represent the group most vulnerable to AI-induced insecurity.

### 4.4 Limitations and future research

Despite the theoretical and practical insights offered by this study, several limitations should be noted to guide future research.

One primary limitation concerns generalizability. The data for this research were collected exclusively from China’s cross-border e-commerce sector. While focusing on a single high-velocity industry enhances internal validity by reducing contextual noise, it inevitably restricts the extent to which these findings can be applied to other sectors or cultural contexts. For instance, the high power distance or collectivist work norms prevalent in China might influence how employees perceive authority and technological change. Consequently, future scholarship would benefit from replicating this model across diverse industries, such as traditional manufacturing or healthcare, and in different cultural settings to test the universality of the suppression mechanisms identified here.

In addition, there is a measurement limitation regarding the AI Application variable. This study employed the Information Systems Use (ISU) scale. Although this instrument is robust for assessing general technology integration, it was not originally designed to capture the specific affordances of modern Generative AI, such as its high degree of autonomy, creativity, and adaptive learning capabilities. While our validity checks confirmed the structural integrity of the scale, it may not fully reflect the nuanced psychological impacts of interacting with generative agents. We strongly recommend that future researchers develop and validate specialized scales for Generative AI to better capture the intensity of algorithmic autonomy.

Furthermore, the use of a cross-sectional design prevents us from drawing definitive causal inferences. Although we minimized common method bias through a multi-source paired design by collecting independent data from employees and supervisors, we cannot empirically rule out the possibility of reverse causality or dynamic feedback loops. Given that employee adaptation to AI is an evolving process rather than a static event, self-efficacy and job insecurity are likely to fluctuate over time. Therefore, future research should adopt longitudinal or experience-sampling designs to capture the temporal dynamics of the “AI–performance paradox.” Such designs would allow researchers to observe how the “inverted U-shaped” sensitivity shifts over time, helping to distinguish whether AI-induced insecurity acts as a temporary technological shock or a chronic stressor as employees acclimate to continuous technological updates.

Finally, while this study identified job insecurity as a critical mechanism, the psychological impact of AI is likely multifaceted. Therefore, incorporating these factors will capture a wider spectrum of psychological costs and benefits associated with sustained AI adoption. Analyzing these distinct pathways is essential for developing a comprehensive model of both resource gains and losses within the human-AI interaction context. Future inquiries could extend our framework by incorporating additional mediators, such as job crafting or cognitive overload, to provide a more comprehensive understanding of the human-AI relationship.

## Data Availability

The datasets presented in this article are not readily available because they contain information that could compromise the privacy of research participants. Requests to access the datasets should be directed to XL, 24081260@siswa.um.edu.my.
